# Photobiomodulation Enhances Tendon Regeneration: A Systematic Review and Meta‐analysis of Preclinical Studies

**DOI:** 10.1002/ars2.70029

**Published:** 2026-07-08

**Authors:** Yose Waluyo, Diah Nurul Islami Muchsin, Muthiah Nur Afifah, Yaldi Rosadi, M. Salas Al Aldi

**Affiliations:** ^1^ Department of Physical Medicine and Rehabilitation, Faculty of Medicine Hasanuddin University Makassar Indonesia; ^2^ Bachelor of Medicine Department, Faculty of Medicine Hasanuddin University Makassar Indonesia

## Abstract

**Purpose:**

To evaluate the preclinical evidence supporting the efficacy of photobiomodulation therapies in tendon regeneration.

**Methods:**

A comprehensive literature search was conducted in MEDLINE, ScienceDirect, and Google Scholar to identify in vivo animal studies evaluating photobiomodulation therapies for tendon healing. Quantitative meta‐analyses were performed for homogeneous outcomes using a random‐effects model.

**Results:**

Out of 4946 screened articles, 36 met the inclusion criteria. Pooled analyses revealed that photobiomodulation significantly downregulated proinflammatory IL‐1β (mean difference [MD]  = −46.14 pg/mL; 95% confidence interval [CI] = −66.45 to −25.84; *P* < .00001) and upregulated anti‐inflammatory IL‐10 (MD = +49.14 pg/mL; 95% CI = 37.41‐60.86; *P* < .00001), indicating a bidirectional immunomodulatory effect. Histologically, photobiomodulation increased the collagen type I/III ratio (standardized MD = +3.0; 95% CI = 2.4‐3.6). Functionally, load‐bearing strength improved significantly compared with controls (MD = +10.07 N; 95% CI = 3.01‐17.13; *P* = .005) with photobiomodulation (low‐level laser therapy and light‐emitting diode), whereas photochemical tissue bonding showed higher tensile strength (MD = +0.57 MPa; 95% CI = 0.37‐0.78; *P* < .00001), reflecting enhanced early mechanical stabilization through sutureless photochemical repair.

**Conclusions:**

Photobiomodulation therapies promote tendon regeneration in both acute and chronic injury models, enhancing tendon regeneration at molecular, histological, and functional levels.

**Clinical Relevance:**

These findings support the potential of photobiomodulation therapies, providing a foundation for clinical trials in human populations to refine rehabilitation protocols and improve musculoskeletal outcomes.

## Graphical abstract

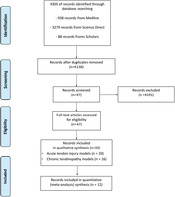

Tendon disorders include traumatic injuries, such as tendon ruptures, and tendinopathy of chronic, inflammatory, and degenerative origin.^1^, ^2^ These conditions are highly prevalent, accounting for nearly half of all musculoskeletal injuries encountered in clinical practice. Achilles tendinopathy is among the most frequently diagnosed forms, with an incidence of 1.8 per 1000 person‐years in the general population due to its critical biomechanical role and exposure to repetitive loading.^3^ Other common disorders include rotator cuff tendinopathy, which is prevalent among overhead athletes and older adults; patellar tendinopathy, affecting up to 24.8% of volleyball players and 20.8% of basketball players; and lateral epicondylitis, with a reported prevalence of 23.4% in high‐risk occupations such as butchery, closely linked to repetitive and forceful upper‐limb tasks.^4^, ^5^, ^6^ Current management strategies for tendon injuries include both conservative and surgical interventions.^7^ However, conservative approaches are often less successful, with only 60% of tendons restored to function and up to 29% of patients requiring surgery after conservative treatment failure.^2^ Although surgical repair may address structural deficits, it carries inherent risks, including iatrogenic trauma, compromised vascularity, inflammation due to foreign body response, adhesion formation, and long‐term functional impairment.^8^ Moreover, the healing process in both acute and chronic tendon pathologies is often incomplete, reflecting the intrinsic limitations of the tendon tissue, such as hypocellularity and poor regenerative capacity.^9^ In addition to conventional surgical repair, several novel light‐based approaches such as photobiomodulation therapy (PBM) and photochemical tissue bonding (PTB) have been proposed to enhance tendon healing and minimize suture‐related complications. Recent systematic evidence has revealed that PTB using Rose Bengal shows favorable histological and functional outcomes as a sutureless repair strategy.^10^ This reinforces the rationale for further investigating the broader photobiomodulatory mechanisms underlying tendon regeneration.

Tendon repair follows 3 overlapping phases (inflammation, proliferation, and remodeling) regulated by cytokines, growth factors, and extracellular matrix signaling. PBM exerts its effects through a photochemical mechanism, in which photons are absorbed by cytochrome c oxidase in mitochondria, enhancing adenosine triphosphate production and reducing nitric oxide‐mediated inhibition. These processes promote cellular metabolism and attenuate excessive inflammation, establishing a biologic rationale for PBM in tendon healing.^11^

These challenges highlight the importance of developing adjunctive modalities that promote tendon healing at both the molecular and cellular levels. PBMs have emerged as promising noninvasive options to complement or substitute surgical treatments. Their ability to modulate cellular repair pathways—particularly through PBM—has positioned them as promising tools in musculoskeletal rehabilitation, especially for tendinopathies where standard interventions often fail to restore structural integrity and biomechanical function.^11^

Current evidence is scattered across animal studies with varying methodologies. A previous systematic review evaluated the effects of low‐level laser therapy (LLLT) on tendon healing.^12^ However, it did not include other PBMs, such as high‐intensity laser therapy, light‐emitting diode (LED), or PTB. It also lacked analysis of molecular mechanisms and did not differentiate between acute and chronic models, providing minimal translational relevance for rehabilitation. The purpose of this study was to evaluate the preclinical evidence supporting the efficacy of PBMs in tendon regeneration. We hypothesized that PBM enhances tendon healing through modulation of molecular pathways, histological remodeling, and restoration of biomechanical function.

## METHODS

We systematically reviewed the relevant documents using the Preferred Report List for Systematic Reviews and Meta‐Analysis Criteria (PRISMA) and registered this study in PROSPERO (Trial registration: PROSPERO [CRD42022363254]).

### Eligibility Criteria

Our systematic review followed the PRISMA recommendations. The acceptable research criteria based on the PICOS approach (PRISMA‐P, 2016) including (**P**) in vivo or ex vivo animal studies evaluating tendon repair using laser as the primary intervention, (**I**) laser or light‐based therapy with a clearly specified wavelength, (**C**) comparison with conventional tendon repair methods, (**O**) assessment of biological markers and functional and/or histologic outcomes, and (**S**) animal and laboratory studies.

The following studies were excluded: review articles, conference abstracts, guidelines, chapter books, perspective papers, abstract‐only articles, non‐English full texts, and those applying experimental approaches to tendon repair using nonlaser modalities, in vitro (cell) experiments, and silico experiments without tissue testing.

### Search Strategy

A comprehensive literature search was conducted in the MEDLINE, ScienceDirect, and Google Scholar databases from inception through June 2025, using appropriate keywords for each database. All authors approved the search strategy, and the following keywords were applied, including for laser (Laser OR Light OR Low‐level laser therapy OR Photoactivated OR Photobiomodulation OR Photochemical tissue bonding), for tendon (Tendon NOT Eye NOT Cornea NOT Nerve NOT Vascular NOT Skin NOT Intestine), for injury (Injury OR Rupture OR Tendinopathy OR Tendinitis), and for repair (Repair OR Welding OR Bonding). The selection process began with removing duplicates and selecting titles and abstracts by 3 reviewers (M.N.A., Y.R., D.N.I.). The full text was obtained when eligibility could not be determined solely from the title and abstract of the article. Studies deemed ineligible per the exclusion criteria on full‐text review were omitted. Disagreements were resolved by joint discussion, and if a consensus could not be reached, a vote was conducted after consultation with a fourth and fifth reviewer (Y.W., M.S.A.A.). Author cooperation was critical for selecting eligible articles, determining article quality, and regularly evaluating the review process. In all cases, the full text was obtained if insufficient information was present in the title and abstract.

### Data Extraction

Three reviewers (M.N.A., Y.R., D.N.I.) collected data. The extracted data were initially cross‐checked by an additional reviewer (Y.W.). During the revision process, a further verification of the extracted data was performed by M.S.A.A., who also contributed to the meta‐analysis. Data collection was conducted for all enrolled studies, including (1) the author and year of publication, (2) study design, (3) population, (4) the total number of samples, (5) interventions, (6) the type of tendons involved, (7) the type of irradiation applied, (8) the observation time, and (9) patient outcomes. Information regarding the sex of animal subjects was not consistently reported across studies and was therefore not extracted or analyzed in this review. This limitation is acknowledged and discussed in the interpretation of results.

A quantitative synthesis was performed for homogeneous outcome domains using Review Manager 5.4 (Cochrane Collaboration). Mean and standard deviation values were extracted from studies reporting comparable parameters. Pooled effect sizes were calculated as the mean difference (MD) or standardized MD (Hedges’ g) under a random‐effects model, with 95% confidence intervals (CIs).

The findings were extracted after selecting the appropriate article, and the data were presented in a Microsoft Word template. The selected search results were exported and compiled using Mendeley citation management software. All data extraction and verification steps were performed independently and cross‐checked among reviewers to ensure consistency.

### Meta‐analysis of Homogeneous Subgroups

A quantitative synthesis was performed for homogeneous outcome domains using Review Manager 5.4 (Cochrane Collaboration). Mean and standard deviation values were extracted from studies reporting comparable parameters. Pooled effect sizes were calculated as the MD or standardized mean difference (Hedges’ g) under a random‐effects model, with 95% CIs.

Heterogeneity was assessed using the *I* ^2^ statistic, where values of 25%, 50%, and 75% represented low, moderate, and high heterogeneity, respectively. Subgroup analyses were conducted according to PBM modality (LLLT vs LED) and healing phase (acute vs subacute). Four primary outcome domains met the homogeneity criteria for meta‐analysis, including functional outcomes such as tensile strength (measured in MPa or Newton), histological outcomes including collagen type I/III ratio or fiber organization, molecular proinflammatory markers such as IL‐1β (pg/mL), and molecular anti‐inflammatory markers such as IL‐10 measured using enzyme‐linked immunosorbent assay.

Data normalization ensured comparable directionality across studies, where higher collagen ratio, IL‐10, and tensile strength indicated improvement and lower IL‐1β indicated reduced inflammation. Publication bias assessment was limited due to the small number of eligible studies per outcome (<10).

### Study Risk of Bias Assessment

The quality assessment was performed using the SYRCLE (Systematic Review Centre for Laboratory Animal Experimentation) risk‐of‐bias tool, which was specifically developed to assess the methodological quality of animal intervention studies.^13^ The transmission problem was formulated to facilitate assessment considering the following parameters: random sequencing, baseline characteristics, allocation concealment, random housing, random caregivers, random result selection, observer blinding, incomplete outcome data, selective reporting, and other biases. The overall risk of bias was classified as unclear, low, or high; a study was considered low‐risk only if all domains for this outcome were assessed as low. If at least 1 domain for these results was assessed as ambiguous, but neither was considered high‐risk, the risk was considered moderate (some concern or unclear). Studies were considered high risk when at least 1 aspect of the findings or some domains were deemed unclear and significantly reduced confidence in the results.^14^

Publication bias was assessed through visual inspection of funnel plot symmetry for each meta‐analyzed outcome (functional, histological, and molecular) using Review Manager version 5.4. Asymmetry was interpreted cautiously due to the limited number of included studies per outcome, following Cochrane Handbook recommendations.

## RESULTS

### Study Selection

**FIGURE 1:**
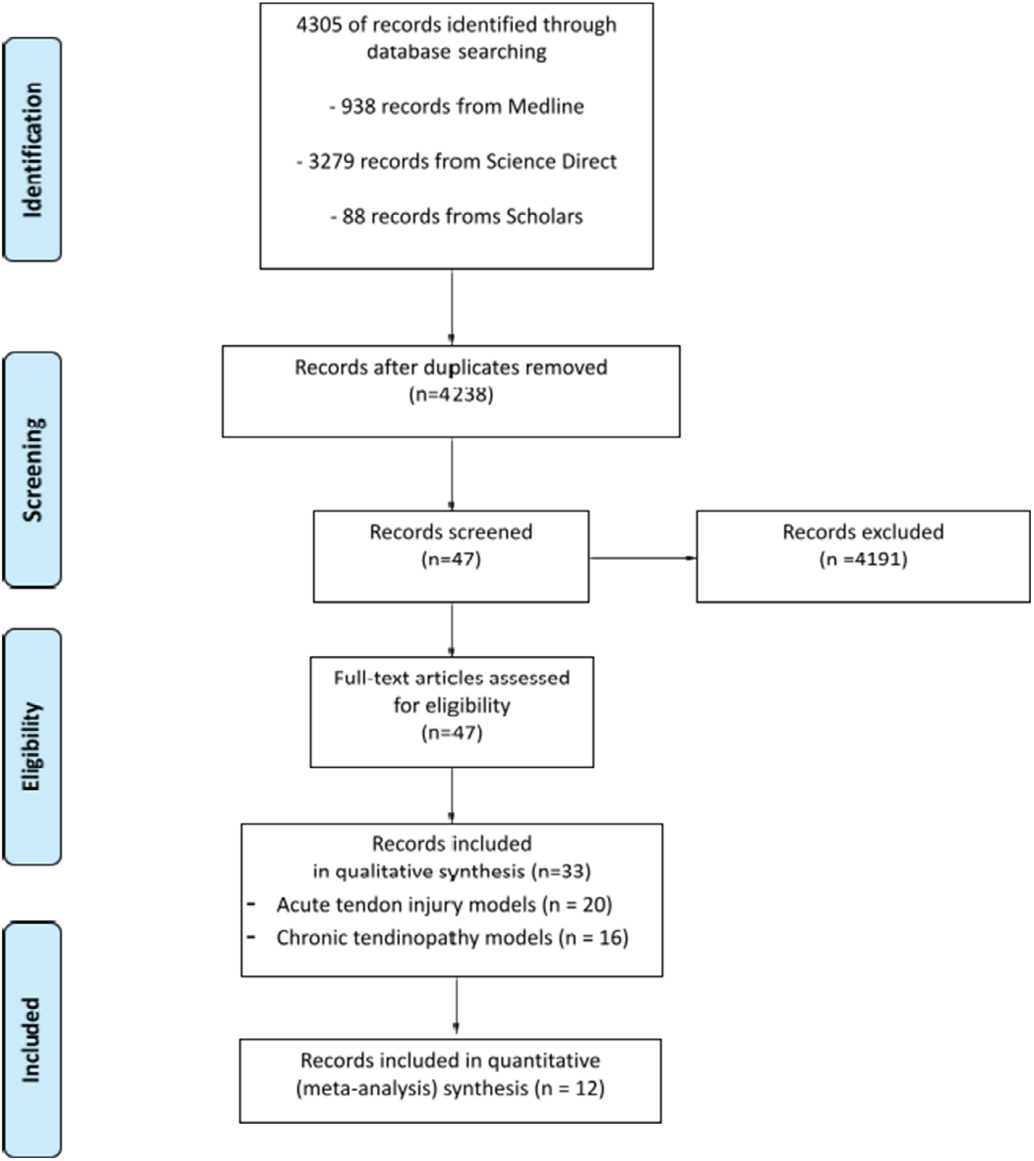
PRISMA 2020 flow diagram illustrating study selection. The final included studies (n = 36) were subcategorized into acute tendon injury models (n = 20) and chronic tendinopathy models (n = 16). A subset of 12 studies met the criteria for quantitative meta‐analysis. (PRISMA, Preferred Report List for Systematic Reviews and Meta‐Analysis Criteria.)

The study selection process is illustrated in the PRISMA study flow diagram (Figure 1). A total of 4946 relevant studies were initially identified through database searches. After removal of duplicates, 4238 records remained for screening. Based on title and abstract assessment, 4191 records were excluded for not meeting the inclusion criteria. Subsequently, 47 full‐text articles were evaluated for eligibility. Of these, 36 studies were included in the qualitative synthesis, comprising 20 acute tendon injury models and 16 chronic tendinopathy models. Among these, 12 studies met the homogeneity criteria and were included in the quantitative synthesis (meta‐analysis).

### Characteristics of Included Studies

**FIGURE 2:**
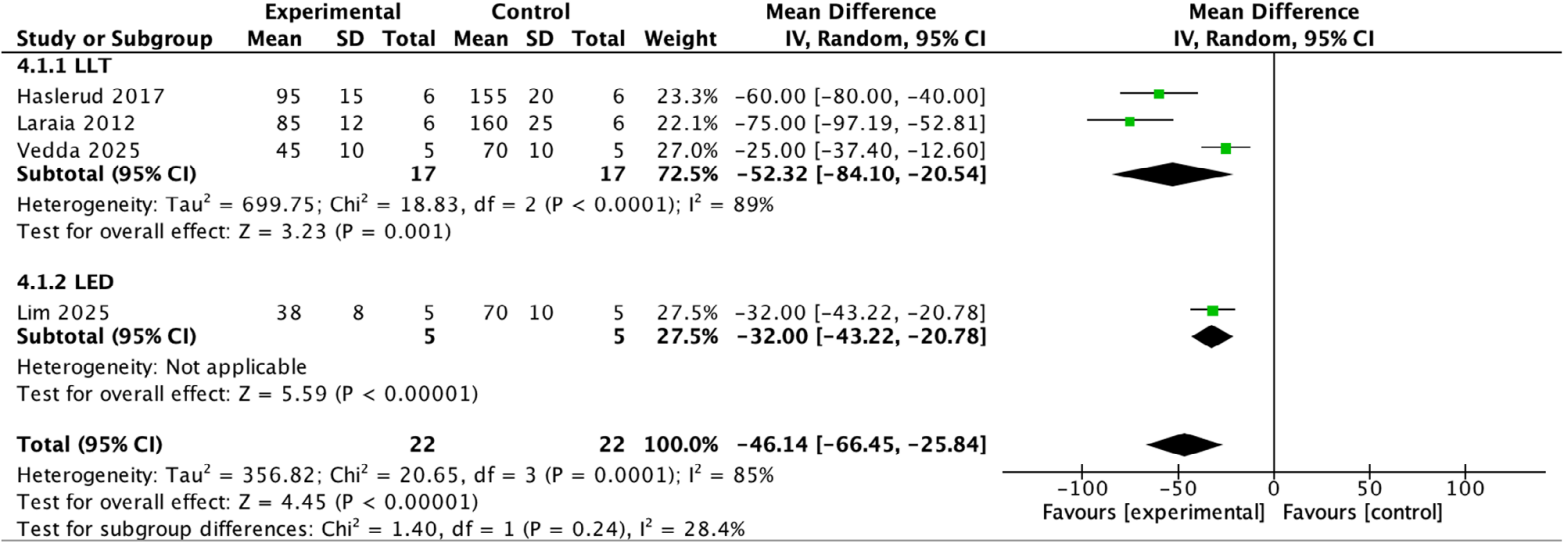
Forest plot of molecular outcome (IL‐1β expression). Meta‐analysis of IL‐1β protein levels showing a significant reduction after photobiomodulation (MD = −46.14 pg/mL; 95% CI −66.45 to −25.84; *P* < .00001). Subgroup analysis revealed similar anti‐inflammatory effects between laser and LED groups, supporting PBM's ability to suppress proinflammatory cytokine activity during tendon healing. (CI, confidence interval; LED, light‐emitting diode; LLT, low‐level laser therapy; MD, mean difference; PBM, photobiomodulation therapy; SD, standard deviation.)

**FIGURE 3:**
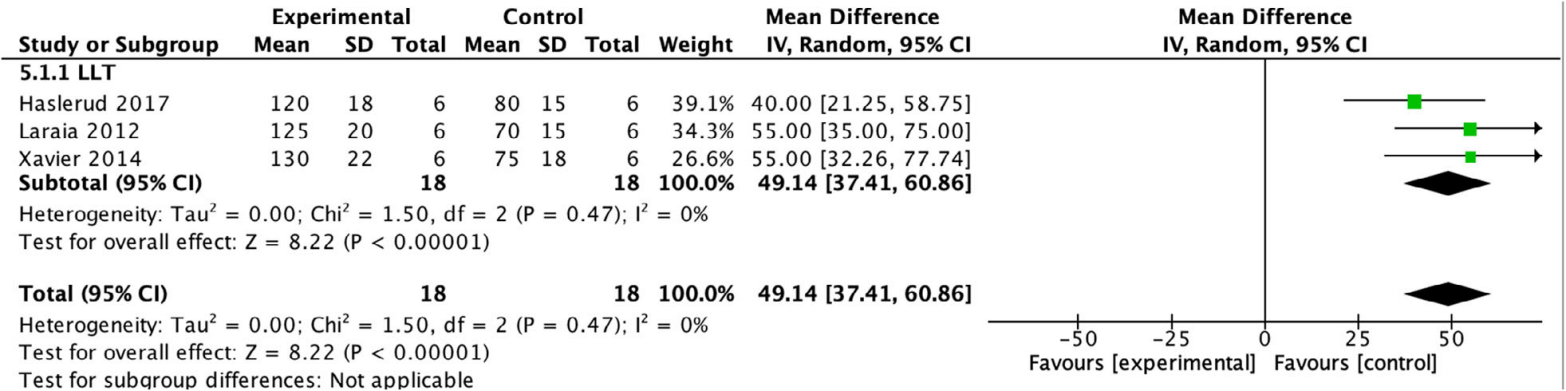
Forest plot of molecular outcome (IL‐10 expression). Meta‐analysis of ELISA‐based IL‐10 data showing a significant increase in anti‐inflammatory cytokine expression following PBM (MD = 49.14 pg/mL; 95% CI 37.41‐60.86; *P* < .00001; *I* ^2^ = 0%). This result highlights PBM's consistent immunomodulatory effect, promoting inflammatory resolution and progression toward tissue regeneration. (CI, confidence interval; ELISA, enzyme‐linked immunosorbent assay; LLT, low‐level laser therapy; MD, mean difference; PBM, photobiomodulation therapy; SD, standard deviation.)

**FIGURE 4:**
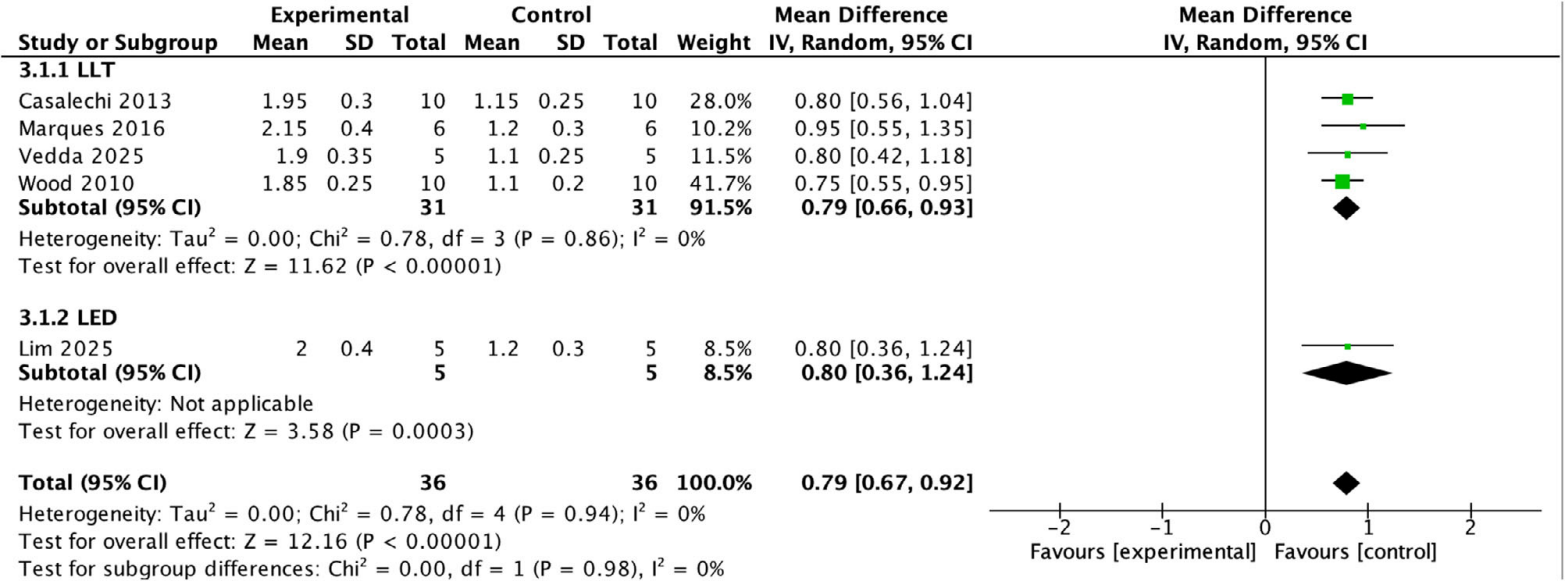
Forest plot of histological outcome (collagen I/III ratio). Pooled analysis of collagen I/III ratio as a marker of tissue remodeling and maturation. PBM significantly enhanced collagen organization and increased the type I/III ratio compared with untreated tendons (SMD = 0.79; 95% CI 0.67‐0.92; *P* < .00001; *I* ^2^ = 0%). These findings indicate accelerated collagen realignment and histological repair quality under PBM exposure. (CI, confidence interval; PBM, photobiomodulation therapy; SD, standard deviation; SMD, standardized mean difference.)

**FIGURE 5:**
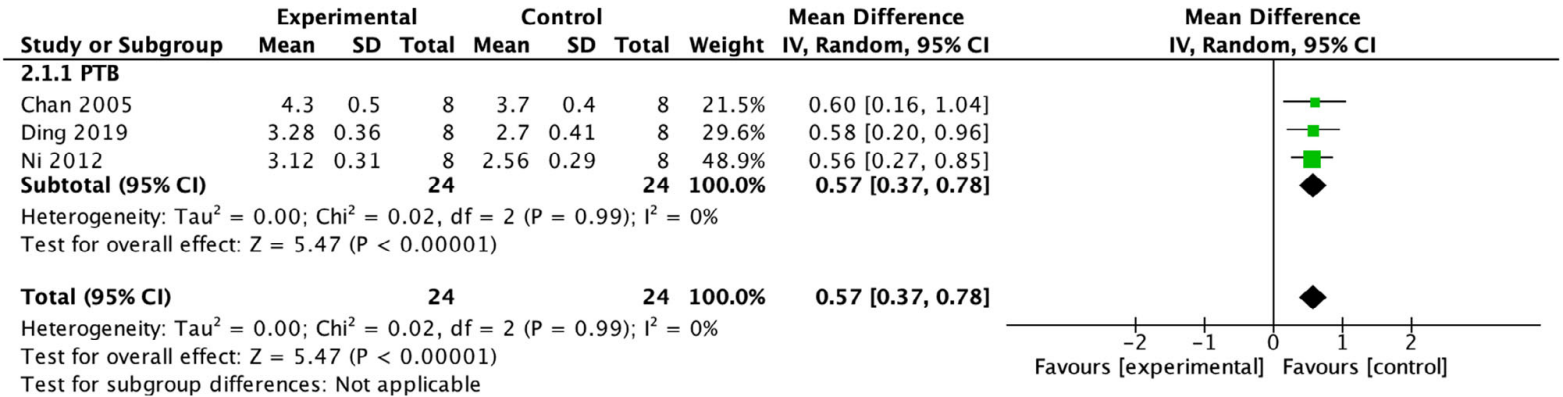
Forest plot of functional outcome (tensile load, N). Meta‐analysis of photobiomodulation (LLLT and LED) studies measuring maximum load to failure in Newtons (N). PBM significantly improved tendon load‐bearing capacity compared with control (pooled MD = 10.07 N; 95% CI 3.01‐17.13; *P* = .005). Although moderate heterogeneity was observed (*I* ^2^ = 87%), the consistent effect direction supports PBM's efficacy in enhancing tensile resilience and structural integrity of healing tendons. (CI, confidence interval, LED, light‐emitting diode; LLLT, low‐level laser therapy; MD, mean difference; PBM, photobiomodulation therapy; PTB, photochemical tissue bonding; SD, standard deviation.)

**FIGURE 6:**
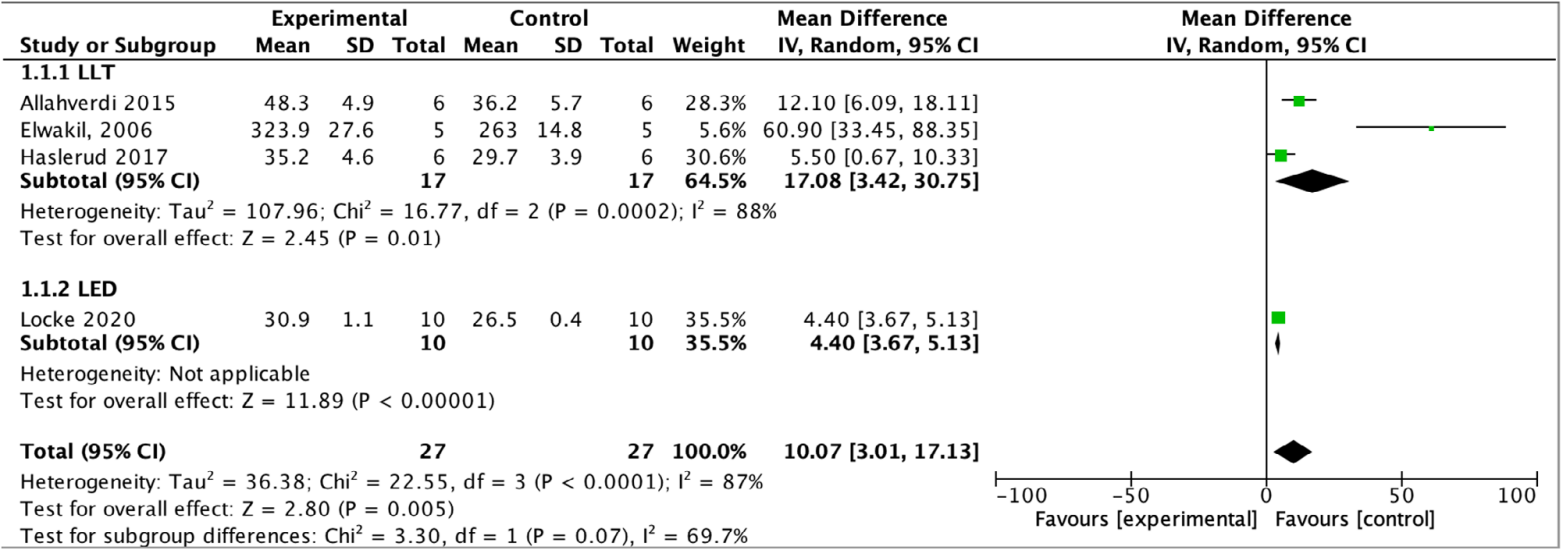
Forest plot of functional outcome (tensile strength, MPa). Meta‐analysis of PTB studies evaluating tendon tensile strength, expressed in MPa. PTB significantly increased tensile strength compared with control groups (pooled MD = 0.57 MPa; 95% CI 0.37‐0.78; *P* < .00001; *I* ^2^ = 0%). These results indicate that PTB enhances early mechanical stabilization and promotes stronger tissue repair through light‐activated collagen cross‐linking. (CI, confidence interval; LED, light‐emitting diode; LLT, low‐level laser therapy; MD, mean difference; MPa, megapascals; PTB, photochemical tissue bonding; SD, standard deviation.)

The main characteristics of the included studies are summarized in Table 1 (acute tendon rupture) and Table 2 (chronic tendinopathy models). Thirty‐six experimental animal studies published between 2005 and 2025 investigated the effects of PBM on tendon healing. Among these, 12 studies fulfilled the homogeneity criteria and were pooled for quantitative meta‐analysis (Figures 2, 3, 4, 5, 6).

**TABLE 1:** Characteristics of Included Animal Studies in Acute Tendon Rupture Models

Author, Year	Animal Species	Study Design	Number of Samples	Intervention	Type of Tendon	Irradiation	Time of Observation	Outcome		
								Biological Marker	Histological Findings	Functional
Locke et al., 2020^15^	Mice	True experimental study	37	G1: PBM G2: control	Achilles tendon	Continuous wave 980 and 810 nm (near infrared) Energy density 2.5 J/cm^2^ Power density 30 or 300 mW/cm^2^ Spot size 1.5 cm^2^	7 wk	‐	‐	Stiffness Male: G2 > G1 (NS) Female: G1 > G2 (Sig) G1 female > G1 male (NS)
										Young's modulus Male: G2 > G1 (NS) Female: G1 > G2 (NS) G1 female > G1 male (Sig)
										Ultimate strain Male: G1 > G2 (Sig)
										Female: G2 > G1 (NS) G1 Female > G1 Male (Sig)
										Ultimate load Male: G1 > G2 (NS) Female: G1 > G2 (NS) G1 Female > G1 Male (NS)
										Ultimate stress Male: G2 > G1 (NS) Female: G1 > G2 (NS) G1 Female > G1 Male (NS)
Fillipin et al., 2005^16^	Wistar rats	True experimental study	32	G1: control G2: trauma G3: trauma + LLLT for 14 d G4: trauma + LLLT for 21 d (L21)	Achilles tendon	Wavelength 904 nm (Ga‐As) Continuous power 45 mW Energy density 5 J/cm^2^	Day 14, 21	Collagen G2 > G4 > G3 > G1 G2 > G1 (Sig) G2 > G3 (Sig) G2 > G4 (Sig)	‐	‐
								SOD G4 > G3 > G2 > G1 G3 > G2, G3 > G1 (Sig) G4 > G2, G1 (Sig)		
								TBARS G2 > G4 > G3 > G1 (Sig)		
								QL G2 > G3 > G4 > G1 (Sig)		
Oliveira et al., 2009^17^	Wistar rats	True experimental study	60	G1: standard G2: control	Calcaneal tendon	Wavelength 830 nm (GaAsAl) Continuous power 40 mW Total energy 0.12 J Spot size 0.028 cm^2^	Days 3, 5, and 7	‐	Collagen fiber G1 > G4 > G5 > G3 > G2 G3 < G5 (NS)	‐
				G3: trauma + LLLT for 3 d G4: trauma + LLLT for 5 d G5: trauma + LLLT for 7 d						
Wood et al., 2010^18^	Wistar rats	True experimental study	50	G1: control, received no treatment G2: US alone G3: LLLT alone G4: US followed by LLLT G5: LLLT followed by US	Calcaneal tendon	Wavelength 830 nm (GaAsAl) Power 40 mW Total energy 0.12 J Spot size 0.028 cm^2^	Day 5	‐	Type 1 collagen G5 > G4 > G2 > G3 > G1 G2 > G1 (Sig) G3 > G1 (Sig) G > G1 (Sig)	‐
									Type 3 collagen G1 > G2 > G4 > G3 > G5 (Sig)	
de Freitas Dutra Júnior et al., 2022^19^	Wistar rats	True experimental study	84	G1: control G2: HFB G3: PBM G4: HFB + PBM	Achilles tendon	Wavelength 660 nm (InGaAlP) Power 40 mW Total energy 0.23 J Spot size 0.04 cm^2^	Days 7, 14, and 21	Collagen quantification Day 7: G3 > G4 > G2 > G1 (NS) Day 14: G3 > G2 > G4 > G1 (NS) Day 21: G4 > G3 > G2 > G1 (NS)	Bonar histological score Day 7: G3 > G1 > G4 > G2 G3 > G1 (Sig) G4 > G2 (Sig) G3 > G2 (Sig) G3 > G4 (Sig) Day 14: G1 > G3 > G2 > G4 G1 > G3 (Sig) G2 > G4 (NS) Day 21: G1 > G3 > G4 > G2 G1 > G3 (Sig) G4 > G2 (NS)	‐
Allahverdi et al., 2015^20^	White New Zealand rabbits	True experimental study	18	G1: control negative (saline) G2: PRP only G3: LLLT only G4: LLLT + PRP	Achilles tendon	Wavelength 650 nm (InGaAlP) Continuous power 30 mW Energy density 1.8 J/cm^2^ Spot size 1 cm^2^	Intervention: days 1, 8, and 15	Hydroxyproline content G5 > G1, G2, G3, G4 (Sig**)	Collagen fibers G1 < G2, G3, G4 (Sig**) G4 > G3, G2 (Sig**) G3 > G2 (NS)	Breaking strength G1 < G2, G3, G4 (Sig**) G4 > G2, G3 (Sig**) G3 < G2 (NS)
							Sacrifice: day 31			
								Healed tendon G1 < G3, G2, G4 (Sig**) G4 > G3, G2 (Sig**) G3 > G2 (NS)		
										Tensile strength G1 < G2, G3, G4 (Sig**) G4 > G2, G3 (Sig**) G3 < G2 (NS)
Laraia et al., 2012^21^	Wistar rats	True experimental study	30	G1: control G2: LLLT	Achilles tendon	Wavelength 660 nm (InGaAlP) Power 100 mW Energy 6 J Spot size 0.028 cm^2^	6, 24, 72 h	IL‐6 6 h: G1 > G2 (NS) 24 h: G1 > G2 (NS) 72 h: G1 > G2 (Sig*) LLLT: 6 h > 24 h > 72 h	Inflammatory infiltrate and cell proliferation 72 h after injury G2 > G1	‐
								IL‐1β 6 h: G1 > G2 (Sig*) 24 h: G1 > G2 (Sig*) 72 h: G1 > G2 (Sig*) LLLT: 6 h < 24 h > 72 h		
								IL‐10 6 h: G1 < G2 (Sig*) 24 h: G1 < G2 (Sig*) 72 h: G1 < G2 (Sig**) LLLT: 6 h < 24 h > 72 h		
Chan et al., 2005^22^	Sprague‐Dawley rat	True experimental study	48	G1: SR (control) G2: RB G3: laser G4: PTB	Achilles tendon	Wavelength 514 nm Energy density 120 J/cm^2^ Power density 1 mW/cm^2^ Spot size 0.3 cm^2^	Days 7 and 14	‐	‐	Tensile strength Day 7: G4 > G2 > G3 > G1 (Sig*) Day 14: G4 > G1 > G2 > G3 (NS) PTB: day 14 > day 7 > day 0 (Sig**)
Ding et al., 2019^23^	Subei chickens	True experimental study	24	G1: HAM/PTB G2: SR G3: control	Flexor digitorum profundus tendon	Wavelength 532 nm Energy density 200 J/cm^2^ Power density 0.5 mW/cm^2^ Spot size 1 cm^2^	Days 3, 7, 14, and 28	‐	Inflammatory sign Day 7: G2 > G1 (Sig) Day 14: G2 > G1 (Sig)	Tensile strength G3 > G2 or G1 (Sig) Day 3: G2 > G1 (NS) Day 7: G2 > G1 (NS) Day 14: G2 < G1 (NS) Day 28: G2 > G1 (NS)
										Joint activity Day 3, 7, 28: G1 > G2 (NS) Day 14: G1 > G2 (Sig)
										Tendon adhesion score Day 3, 7, 28: G2 > G1 (NS) Day 14: G2 > G1 (Sig)
Zhu et al., 2020^24^	Sprague‐Dawley rat	Quasiexperimental study	4	G1: Control G2: SR G3: PTB G4: UCNPs + 808 nm	Achilles tendon	Wavelength 532 nm and 808 nm Spot size 1.5 cm^2^	Days 7 and 14	‐	Cell apoptosis Control: day 7 < day 14 (NS) SR: day 7 > day 14 (Sig) PTB: day 7 > day 14 (Sig) UCNPs + 808 nm: day 7 > day 14 (Sig) Day 7: G2 > G3 > G4 (Sig) Day 14: G2 > G3 > G4 (Sig) Day 14: G2 > G3 (Sig)	‐
									Fiber collagen Day 7: G1 < G2, G3 and G4 Day 14: G2 < G3 and G4	
Yao et al., 2018^25^	‐	True experimental study	12	G1: control (SR) G2: PTB 20 s G3: PTB 40 s G4: PTB 80 s G5: PTB 120 s G6: PTB 200 s	Achilles tendon	Energy density 108 J/cm^2^ Power density 0.3 W/cm^2^ Spot size 1.5 to 2 cm^2^	Days 7, 14, and 28	Cell proliferation 24 h: G5 > G1 (Sig) 48 h: G4 > G1 (Sig), G1 > G6 (Sig) 72 h: G4 > G1 (Sig), G1 > G6 (Sig)	‐	‐
								ROS G4 > G5 > G3 > G2 > G1 > G6 (Sig)		
								Injured tenocytes migration G4 > G5 > G1 > G2 > G3 > G6 G4 > G1 (Sig) G5 > G1 (Sig) G1 > G6 (Sig)		
Da Ré Guerra et al., 2016^26^	Wistar rats	True experimental study	85	G1: intact/control G2: injury G3: injury + LLLT (continuous) G4: injury + LLLT (pulsed laser)	Achilles tendon	Wavelength 830 nm (GaAsAl) Energy density 4 J/cm^2^ Power 40 mW	Days 1, 4, 8, and 15	Proinflammatory cytokines TNF‐α Day 1: NS Day 4: G2 > G1 (Sig), G2 > G4 (Sig), G2 > G3 (NS), G3 > G4 (NS) IL‐1β Day 1: G4 > G2 (Sig), G4 > G1 (Sig), G4 > G3 (Sig), G2 > G3 (NS) Day 4: NS TGF‐β1 Day 8: G4 > G3 (Sig), G4 > G2 (Sig) G4 > G1 (Sig), G3 > G2 (NS) Day 15: G4 > G2 (Sig) G4 > G1 (Sig), G4 > G3 (NS) NO Day 8: G2 > G4 (Sig) G2 > G1 (Sig), G4 > G3 (NS) Day 15: G4 > G2 (Sig) G4 > G1 (Sig) G4 > G3 (Sig), G2 > G3 (Sig) i‐NOS Day 8: day 2 > day 1 (Sig), day 2 > day 4 (Sig), day 3 > day 1 (Sig), day 3 > day 4 (Sig), day 3 > day 2 (NS) Day 15: day 2 > day 1 (Sig), day 4 > day 1 (Sig), day 2 > day 3 (NS), day 4 > day 3 (NS)	‐	‐
Ni et al., 2012^27^	White New Zealand female rabbits	True experimental study	64	G1: ES/PTB G2: SR + ES/PTB G3: SR	Flexor digitorum profundus tendon	Wavelength 532 nm Energy density 108 J/cm^2^ Power density 0.3 mW/cm^2^ Spot size 1 cm^2^	Days 7, 14, and 28	‐	‐	Ultimate stress Day 7: G2 > G3 > G1 (Sig) G3 > G1 (NS) Day 14: G2 > G3 > G1 (Sig) G3 > G1 (NS) Day 28: G3 > G1 (NS)
										Young's modulus Day 7: G2 > G3 > G1 (NS) Day 14: G2 > G3 > G1 (NS) Day 28: G3 > G1 (NS)
										Tendon adhesion Score Day 7: G3 > G1 > G2 (Sig) Day 14: G3 > G1 = G2 (Sig) Day 28: G3 > G1 (Sig)
Da Ré Guerra et al., 2013^28^	Wistar	True experimental study	140	G1: intact G2: injured G3: injured + continuous LLLT G4: injured + pulse LLLT G5: injured G6: injured + continuous LLLT G7: injured + pulse LLLT (20 Hz until the 7th day and 2 kHz from 8 to 14 d) G2, G3, G4: euthanized day 8 G5, G6, G7: euthanized day 15	Achilles tendon	Wavelength 830 nm (GaAsAl) Energy density 4 J/cm^2^ Power 40 mW	Days 7 and 14	Hydroxyproline G1 > G7 > G6 > G5 > G2 > G4 > G3 G1 > G2 (Sig), G1 > G3 (Sig), G1 > G4 (Sig), G1 > G5 (Sig), G1 > G6 (Sig)	‐	‐
								Noncollagenous protein G4 = G5 > G6 > G3 > G7 > G2 > G1 G1 < G2 (Sig), G1 < G3 (Sig), G1 < G4 (Sig), G1 < G5 (Sig), G1 < G6 (Sig)		
								Western blotting collagen 1		
								G7 > G4 > G1 > G6 > G3 > G5 > G2		
								Western blotting collagen 3 G4 > G5 > G3 > G2 > G6 > G1 > G7		
Elwakil et al., 2007^29^	Rex rabbits	True experimental study	30	G1: He‐Ne laser G2: SR	Achilles tendon	Continuous wavelength 632.8 nm (He‐Ne) Energy density 1 J/cm^2^	Days 1, 5, and 14	‐	‐	UTS G1 > G2 (Sig) Load at break G1 > G2 (Sig)
										Extension at break G1 > G2 (NS) Energy to failure G1 > G2 (Sig)
Barbosa et al., 2013^30^	Wistar rats	True experimental study	54	G1: injured G2: PRP G3: LLLT 660 nm G4: LLLT 830 nm G5: PRP + LLLT 660 nm G6: PRP + LLLT 830 nm	Calcaneal tendon	Wavelength 660 (InGaAlP) and 830 nm (GaAlAs) Energy 0.2 J Power 100 mW Spot size 0.28 cm^2^	Day 13	Type 1 collagen G6 > G2 > G1 (Sig) G6 > G3 > G2 (Sig) G4 > G3 (NS) G6 > G5 > G4 > G3 > G2 (Sig) G6 > G5 (Sig)	‐	‐
								Type 3 collagen G1 > G2 > G6 (Sig) G3 > G2 > G6 (Sig) G4 > G3 > G2 > G5 > G6 (Sig) G4 > G3 (NS) G5 > G6 (NS)		
Carrinho et al., 2006^31^	Wistar rat	True experimental study	48	G1: 685 nm laser (3 J/cm^2^) G2: 685‐nm laser (10 J/cm^2^) G3: 830‐nm laser (3 J/cm^2^) G4: 830‐nm laser (10 J/cm^2^) G5: injured control G6: noninjured control	Achilles tendon	Wavelength 685 (InGaAlP) and 830 nm (GaAlAs) Energy density 5.4 J/cm^2^ Power 15 mW Spot size 0.0028 cm^2^	Day 13	Collagen fiber G6 > G1 > G3 > G2 > G4 > G5 G1 > G2 (Sig) G1 > G3 (Sig) G1 > G4 (Sig) G3 > G2 (NS) G2 > G4 (Sig) G3 > G4 (Sig)	‐	‐
Nicodemo et al., 2024^32^	Wistar rats	True experimental study	75	G1: control (C) G2: injury (I) G3: injury + LLLT (LA) G4: injury + HAM (AM) G5: injury + laser + HAM (LAM)	Achilles tendon	Wavelength 660 nm Continuous power 40 mW Total energy 0.4 J Energy density 1 J/cm^2^ Spot size 4 mm^2^	Days 3, 7, and 14	‐	Collagen fiber organization Day 3: G1 > G5, G4, G3, G2 Day 14: G1 > G5, G4 > G3 > G2 Day 14: G1 > G5 > G4, G3 > G2	‐
									Inflammatory cell Day 14: G2 > G1 > G3 > G5 > G4 G2 > G1 (Sig) G2 > G3 (Sig) G2 > G4 (Sig) G2 > G5 (Sig)	
									Tenocytes Day 14: G5 > G3 > G4 > G2 G3 > G1 (Sig) G5 > G1 (Sig) G5 > G2 (Sig) G5 > G4 (Sig)	
Lim et al., 2025^33^	Mice	True experimental study	40	G1: intact G2: injured + LED nonirradiated G3: injured + LED irradiated	Achilles tendon	Wavelengths of 630 nm power 10 mW/cm^2^ Energy densities 12 J/cm^2^ Wavelengths 880 nm Power 40 mW/cm^2^ Energy densities 48 J/cm^2^	Day 14	Tnmd G2 > G3 > G1 G2 > G3 (Sig) G2 > G1 (NS)	Fiber structure and fiber arrangement G2 > G3 > G1 (Sig)	‐
								SCX, TGF‐β1 and vimentin G2 > G3 > G1 G2 > G3 (NS) G2 > G1 (Sig)	Cell density and roundness of the nuclei G3 > G2 > G1 (NS)	
								Type 1 and 3 collagen, M2 macrophage G2 > G3 > G1 G2 > G3 (Sig) G2 > G1 (Sig)		
								Col1/Col3 ratio G3 > G1 > G2 G3 > G2 (Sig) G1 > G2 (NS)		
								Pan macrophage G3 > G2 > G1 (NS)		

*Note:* Sig* and Sig** indicate varying levels of statistical significance as reported in the original studies.

‐, not available; AM, amniotic membrane; Col, collagen; d, day; ES, electrospun silk mat; h, hour; HAM, human amniotic membrane; HFB, heterologous fibrin biopolymer; IL, interleukin; i‐NOS, isoform of nitric oxide synthase; LA, photobiomodulation; LAM, photobiomodulation combined with amniotic membrane; LLLT, low‐level laser therapy; NO, nitric oxide; NS, nonsignificant; PBM, photobiomodulation therapy; PRP, platelet‐rich plasma; PTB, photochemical tissue bonding; QL, hydroperoxide‐initiated chemiluminescence; RB, Rose Bengal; ROS, reactive oxygen species; s, seconds; SCX, scleraxis; Sig, significant; SOD, cytosolic superoxide dismutase; SR, suture repair; TBARS, thiobarbituric acid reactive substance; TGF‐β1, transforming growth factor β1; TNF‐α, tumor necrosis factor α; Tnmd, tenocytes express tenomodulin; UCNPs  + 808, UCNPs@RB‐hydrogel + 808 nm; US, ultrasound; UTS, ultimate tensile strength; wk, week.

**TABLE 2:** Characteristics of Included Animal Studies in Chronic Tendinopathy Models

Author, Year	Animal Species	Study Design	Number of Samples	Intervention	Type of Tendon	Irradiation	Time of Observation	Outcome		
								Biological Marker	Histological Findings	Functional
Marcos et al., 2011^34^	Wistar rats	True experimental study	150	G1: Nacl G2: collagen G3: diclofenac G4: 1 J G5: 3 J G6: 6 J	Achilles tendon	Continuous wave 810 nm (infrared) Energy 1, 3, and 6 J Power 100 mW Spot size 0.028 cm^2^	24 h	Cox‐1 G2 > G5 > G4 > G3 > G6 > G1 G2 > G3 (Sig) Cox‐2 G2 > G3 > G6 > G4 > G5 > G1 G3 > G1 (Sig), G4 > G1 (Sig), G5 > G1 (Sig)	‐	‐
Marcos et al., 2012^35^	Wistar rats	True experimental study	30	G1: control (intact) G2: collagenase G3: diclofenac G4: LLLT 1 J G5: LLLT 3 J	Achilles tendon	Continuous wave 810 nm (infrared) Energy 1 and 3 J Power 100 mW Spot size 0.028 cm^2^	Days 7 and 14	COX‐2 G3 > G2 > G4 > G5 > G1 PGE2 G2 > G5 > G1 > G4 > G3 TNF‐α G2 > G3 > G4 > G5 > G1	‐	‐
								MMP‐3 G2 > G3 > G4 > G5 > G1 MMP‐9 G3 > G2 > G4 > G5 > G1 MMP‐13 G3 > G2 > G5 > G4 > G1		
Marcos et al., 2014^36^	Wistar rats	True experimental study	60	G1: control (noninjured) G2: tendinitis G3: diclofenac G4: LLLT 1 J G5: LLLT 3 J	Achilles tendon	Continuous wave 810 nm (infrared) Energy 1 and 3 J Power 100 mW Spot size 0.028 cm^2^	Day 7	MMP‐3 G2 > G3 > G4 > G5 > G1 G2 > G1 (NS) G2 > G3 > G1 (NS) G2 > G4 > G1 (NS) G2 > G5 > G1 (NS) MMP‐9 G3 > G2 > G4 > G5 > G1 G2 > G1 (NS) G3 > G2 > G4 (NS) G3 > G2 > G5 (NS) MMP‐13 G3 > G2 > G5 > G4 > G1 G2 > G1 (NS) G2 > G4 > G1 (NS) G2 > G5 > G1 (NS)	‐	Stretches G1 > G4 > G2 > G5 > G3 G1 > G2 (NS) G1 > G3 (Sig) G2 > G3 (NS) G1 > G4 > G2 (NS) G1 > G2 > G5 (NS) Forces G1 > G4 > G3 > G5 > G2 G1 > G2 (Sig) G1 > G3 > G2 (NS) G1 > G4 (NS) G4 > G2 (Sig)
Da Ré Guerra et al., 2017^37^	Wistar/Uni rats	True experimental study	15	G1: control (noninjured) G2: injured G3: LLLT 1 and 3 h	Achilles tendon	Wavelength 830 nm (GaAlAs) Energy density 4 J/cm^2^ Power 40 mW	4 h	‐	Collagen G1 > G2 (Sig) G1 > G3 (Sig) Noncollagen G2 > G3 > G1 G2 > G1 (Sig) MMP‐2 G3 > G2 > G1 (Sig) MMP‐9 G3 > G1 (Sig) G2 > G1 (Sig)	‐
Xavier et al., 2010^38^	Rat	True experimental study	56	G1: control (noninjured) G2: injured G3: LEDT therapy for both experimental periods G4: LED therapy 7th to 14th day for 14‐d experimental period	N/A	Wavelength 880 ± 10 nm (LED) Energy density 7.5 J/cm^2^ Power 22 mW Spot size 0.5 cm^2^	Days 7 and 14	Inflammatory cells Day 7: G2 > G3 > G1 G2 > G3 (Sig) Day 14: G2 > G3 > G4 > G1 G2 > G3 (Sig) G2 > G4 (Sig) TNF‐α Day 7: G2 > G3 > G1 G2 > G3 (Sig) Day 14: G2 > G4 > G3 > G1 G2 > G3 (Sig) G2 > G4 (Sig)	Inflammatory cell Day 7: G3 > G2 > G1 Day 14: G2 > G3 > G4 > G1	‐
								IL‐6 Day 7: G2 > G3 > G1 (Sig) Day 14: G2 > G4 > G3 > G1 G3 > G2 > G1 (Sig) G2 > G4 > G1 (Sig) IL‐1β Day 7: G2 > G3 > G1 G2 > G3 (Sig) Day 14: G2 > G4 > G3 > G1 G2 > G3 (Sig) COX2 Day 7: G2 > G3 > G1 (Sig) Day 14: G3 > G2 > G4 > G1 G3 > G1 (Sig) G4 > G1 (Sig)		
Xavier et al., 2014^39^	Wistar rats	True experimental study	30	G1: control (noninjured) G2: injured G3: LEDT (both experimental periods, 14 d) G4: LEDT delay (only 7th to 14th day experimental period)	Achilles tendon	Wavelength 880 ± 10 nm (LED) Energy density 7.5 J/cm^2^ Power 22 mW Spot size 0.5 cm^2^	Days 7 and 14	IL‐10 Day 7: G3 > G2 > G1 G3 > G2 (Sig) Day 14: G4 > G3 > G2 > G1 Collagen I Day 7: G3 > G1 > G2 G3 > G2 (Sig) Day 14: G3 > G2 > G4 > G1 G3 > G1 (Sig) Collagen III Day 7: G3 > G1 > G2 G3 > G2 (Sig) Day 14: G3 > G4 > G1 > G2 G3 > G2 (Sig) G4 > G2 (Sig)	**‐**	‐
Naterstad et al., 2018^40^	Wistar rats	True experimental study	205	G1: control (noninjured) G2: injured	Achilles tendon	Continuous wave 810 nm (infrared) Energy 3 J Power 100 mW Spot size 0.0028 cm^2^	12, 24, 48, 72 h, 5, 7, 14, 21 d	Collagen 12 h G4 > G5 (Sig), G3 > G2 (Sig), G4 > G2 (Sig), G5 > G2 (Sig) 24 h G4 > G5 (Sig), G3 > G2 (Sig), G4 > G2 (Sig), G5 > G2 (Sig) 48 h G3 > G2 (Sig), G3 > G2 (Sig), G4 > G2 (Sig) 72 h G3 > G2 (Sig), G4 > G2 (Sig), G4 > G5 (Sig) 5 d G3 > G2 (Sig), G4 > G5 (Sig) 7 d G3 > G2 (Sig), G4 > G5 (Sig) 14 d G3 > G2 (Sig), G4 > G2 (Sig), G4 > G5 (Sig) 21 d G4 > G5 (Sig)	‐	‐
				G3: LLLT G4: diclofenac (NSAID) G5: dexamethasone (GCS)						
Casalechi et al., 2013^41^	Wistar rats	True experimental study	30	G1: control (noninjured) G2: acute injured G3: chronic injured G4: 780 nm in acute phase and euthanized in day 7 G5: 780 nm in acute phase and euthanized in day 14 G6: 780 nm in chronic phase and euthanized in day 14	Achilles tendon	Wavelength 780 nm (GaAsAl) Energy 1.54 J Power 22 mW Spot size 0.205 cm^2^	Days 7 and 14	IL‐10 G4 > G1 (Sig) G4 > G2 (Sig) G6 > G1 (Sig) G6 > G3 (Sig) G5 > G2 (Sig) G5 > G6 (Sig) VEGF G4 > G2 (Sig) G4 > G1 (Sig) G5 > G3 (Sig) G4 > G1 (Sig) G5 > G1 (Sig) MMP‐1 G2 > G4 (Sig) G3 > G5 (Sig) G6 > G5 (Sig) MMP‐13 G4 > G2 (Sig) G4 > G1 (Sig)	‐	‐
Casalechi et al., 2014^42^	Wistar rats	True experimental study	30	G1: control (noninjured) G2: injured G3: LLLT G4: diclofenac	Achilles tendon	Wavelength 830 ± 10 nm (GaAlAs) Energy 6 J Power 50 mW Spot size 0.028 cm^2^	Days 7 and 14	Inflammatory cells Day 7: G2 > G4 > G3 > G1 G4 > G3 (Sig) G2 > G3 (Sig) G2 > G4 (Sig) Day 14: G2 > G4 > G3 > G1 G2 > G3 (Sig) G2 > G4 (Sig)	Collagen day 7 G1: collagen type 1 > Collagen type 3 G2: collagen type 1 = collagen type 3 G3: collagen type 1 > collagen type 3 G4: collagen type 1 < collagen type 3 Collagen day 14 G3: collagen type 1 > collagen type 3 G4: collagen type 1 = collagen type 3	‐
Torres‐Silva et al., 2014^43^	Wistar rats	True experimental study	30	G1: control (noninjured) G2: Injured G3: diclofenac G4: LLLT 1 J G5: LLLT 3 J	Achilles tendon	Wavelength 660 nm (InGaAlP) Energy 1 and 3 J Power 100 mW Spot size 0.028 cm^2^	2 h	TNF‐α G2 > G4 > G3 > G5 > G1 (Sig) IL‐6 G3 > G4 > G2 > G5 > G1 (Sig) COX2 G3 > G2 > G4 > G5 > G1 (Sig) IL‐10 G3 > G4 > G5 > G2 > G1 (Sig)	‐	‐
Evangelista et al., 2021^44^	Wistar rats	True experimental study	30	G1: control (noninjured) G2: injured G3: LED	Achilles tendon	Wavelength 630 ± 20 nm (LED) Energy 9 J Power 300 mW Spot size 1 cm^2^	24 h	HSCORE for HSP70 G3 > G2 > G1 (Sig)	Fibroblast G1 > G3 > G2 G3 > G2 (Sig) G1 > G2 (Sig) Collagen fiber G3 > G1 > G2 G3 > G2 (Sig) G1 > G2 (Sig)	‐
Pires et al., 2011^45^	Wistar rats	True experimental study	42	G1: control (noninjured) G2: acute injured G3: chronic injured G4: 780 nm in acute phase and euthanized in day 7 G5: 780 nm in acute phase and euthanized in day 14 G6: 780 nm in chronic phase and euthanized in day 14	Achilles tendon	Wavelength 780 nm (GaAsAl) Energy density 7.7 J/cm^2^ Power 22 mW Spot size 0.2 cm^2^	Days 7 and 14	TNF‐α G2 > G1 (Sig) G2 > G4 (NS) G2 > G1 (Sig) G3 > G5 (NS) G3 > G6 (Sig) IL‐1β G2 > G1 (Sig) G2 > G4 (NS) G3 > G1 (Sig) G3 > G4 (NS) G3 > G6 (NS) IL‐6 G2 > G1 (Sig) G2 > G4 (Sig) G2 > G5 (Sig) G3 > G1 (Sig) G3 > G4 (Sig) G3 > G6 (Sig) COX2 G2 > G1 (Sig) G2 > G4 (Sig) G2 > G4 (Sig) G3 > G1 (Sig) G3 > G5 (Sig) G3 > G6 (Sig) TGF‐β G2 > G1 (Sig) G2 > G4 (Sig) G2 > G5 (Sig) G3 > G1 (Sig) G3 > G4 (Sig) G3 > G6 (Sig)	‐	‐
Marques et al., 2016^46^	Wistar rat	True experimental study	42	G1: control (noninjured) G2: injured G3: PBM	Achilles tendon	Wavelength 830 ± 10 nm (GaAsAl) Energy 3 J Power 50 mW Spot size 0.028 cm^2^	Days 7, 14, and 21	MMP‐3 Day 7: G2 > G3 > G1 Day 14: G2 > G3 > G1 Day 21: G2 > G1 > G3 MMP‐9 Day 7: G2 > G3 > G1 Day 14: G2 > G1 > G3 Day 21: G2 > G1 > G3 VEGF Day 7: G3 > G1 > G2 Day 14: G3 > G1 > G2 Day 21: G1 > G3 > G2	Collagen type 1 Day 7: G1 = G3 > G2 Day 14: G1 = G3 > G2 Day 21: G3 > G1 > G2 Collagen type 3 Day 7: G2 > G3 > G1 Day 14: G2 > G1 > G3 Day 21: G2 > G1 > G3	‐
De Jesus et al., 2016^47^	Wistar rat	True experimental study	65	G1: control G2: Sham 1 G3: laser 1 G4: Sham 3 G5: laser 3 G6: Sham 7 G7: laser 7	Achilles tendon	Wavelength 780 nm (GaAsAl) Energy density 17.5 J/cm^2^ Power density 1.75 mW/cm^2^ Spot size 0.04 cm^2^	Days 1, 3, and 7	VEGF Sham: proximal vs intermediate vs distal (NS); laser: proximal vs intermediate vs distal (NS) Sham vs control (NS) Laser vs control (NS) Day 1: G2 < G3 Day 3: G4 < G5 Day 7: G6 < G7	‐	‐
De Jesus et al., 2019^48^	Wistar rat	True experimental study	65	G1: control G2: Sham 1 G3: laser 1 G4: Sham 3 G5: laser 3 G6: Sham 7 G7: laser 7	Achilles tendon	Wavelength 780 nm (GaAsAl) Energy density 17.5 J/cm^2^ Power density 1.75 mW/cm^2^ Spot size 0.04 cm^2^	Days 1, 3, and 7	MMP‐1 Sham: proximal vs intermediate vs distal (NS) Laser: proximal vs intermediate vs distal (NS) G2, G6 vs Control (Sig*) G3, G5, G7 vs control (Sig**) Day 1: G2 > G3 Day 3: G4 > G5 Day 7: G6 > G7 MMP‐3 Sham: proximal vs intermediate vs distal (NS) Laser: proximal vs intermediate vs distal (NS) G2, G4, G6 vs control (NS) G3, G5 vs control (Sig*) Day 1: G2 > G3 Day 3: G4 > G5 Day 7: G6 < G7 MMP‐13 Sham: proximal vs intermediate vs distal (NS) Laser: proximal vs intermediate vs distal (NS) G2, G6 vs control (Sig**) G3, G5, G7 vs control (Sig**) Day 1: G2 > G3 Day 3: G4 > G5 Day 7: G6 < G7	‐	‐

Haslerud et al., 2017^49^	Wistar rats	True experimental study	36	G1: control (noninjured) G2: injured G3: LLLT G4: cryotherapy G5: LLLT + cryotherapy G6: cryotherapy + LLLT	Achilles tendon	Continuous Wave 810 nm (GaAsAl) Energy 3 J Power 100 mW Spot size 0.028 cm^2^	24 h	IL‐1β G2 > G4 > G1 > G5 > G6 > G3 G2 > G1 (Sig) G2 > G3 (Sig) G2 > G5 (Sig) G2 > G6 (Sig) IL‐6 G2 > G1 (Sig) G5 > G2 (Sig) G6 > G2 (Sig) TNF‐α G2 > G1 > G3 > G4 > G5 > G6 G2 > G5 (Sig) G2 > G6 (Sig) IL‐10 G3 > G4 > G5 > G6 > G2 > G1 (NS)	‐	Force G1 > G5 > G4 > G3 > G2 > G6 G1 > G2 (Sig) G3 > G2 (Sig) G6 > G2 (Sig)
Vedda et al., 2025^50^	Wistar rats	True experimental study	53	G1: control (noninjured) G2: injured G3: PBM 660 nm G4: PBM 808 nm	Achilles tendon	Wavelength 660 nm or 808 nm Energy 3 J Power 100 mW Spot size 0.028 cm^2^ Energy density 107.14 J/cm^2^	Day 7	Astrocyte and IL‐1β G2 > G1 > G3 > G4 (Sig)	Collagen type 1 G4 > G1 > G3 > G2 Collagen type 3 G2 > G3 > G4 > G1	‐

*Note*: Sig* and Sig** indicate varying levels of statistical significance as reported in the original studies.

Cox, cyclooxygenase; d, day; GCS, glucocorticoid drugs; h, hour; IL, interleukin; LED, light‐emitting diode; LEDT, light‐emitting diode therapy; LLLT, low‐level laser therapy; MMP, matrix metalloproteinase; N/A, not available; NS, nonsignificant; NSAID, nonsteroid anti‐inflammatory drugs; PBM, photobiomodulation therapy; PGE2, prostaglandin E2; Sig, significant; TGF‐β, transforming growth factor β; TNF‐α, tumor necrosis factor α; VEGF, vascular endothelial growth factor; wk, week.

The included studies used various animal models, predominantly Wistar rats,^16^, ^28^, ^30^ followed by mice,^15^, ^27^ New Zealand white rabbits,^22^, ^23^ Sprague‐Dawley rats,^20^, ^29^ and Subei chickens.^24^ The majority investigated the Achilles tendon, whereas several examined the calcaneal or flexor digitorum profundus tendons. Control groups generally consisted of nonirradiated injured tendons, untreated (intact) controls, or pharmacological comparators such as diclofenac, dexamethasone, or platelet‐rich plasma.

PBMs (LLLT, LED, and PTB) were used in the included studies to manage tendon disorders. The wavelengths applied varied depending on the device type, including gallium arsenide (Ga‐As) lasers at 904 nm; gallium‐aluminum‐arsenide (GaAlAs) lasers at 780, 810, and 830 nm; indium‐gallium‐aluminum‐phosphide (InGaAlP) lasers at 650, 660, and 685 nm; near‐infrared diode lasers at 810 and 980 nm; LEDs at 630 and 880 nm; and helium‐neon (He‐Ne) lasers at 632.8 nm.

For PTB, it used green light with wavelengths of 514 and 532 nm, which had previously been applied with Rose Bengal. Notably, none of the included studies investigated high‐intensity laser therapy, and its effects were not analyzed in this review.

Outcome parameters were classified into biological, histological, and functional domains. Biological markers included collagen types I and III, inflammatory cytokines (IL‐1β, IL‐6, tumor necrosis factor α, and IL‐10), oxidative stress markers (reactive oxygen species, thiobarbituric acid reactive substance, and hydroperoxide‐initiated chemiluminescence), matrix metalloproteinases (MMPs) (1, 3, 9, 13), nitric oxide, vascular endothelial growth factor, transforming growth factor β1 (TGF‐β1), and tendon‐related proteins such as tenomodulin and scleraxis.^16^, ^17^, ^19^, ^20^, ^21^, ^25^, ^26^, ^28^, ^30^, ^31^, ^33^, ^34^, ^35^, ^36^, ^37^, ^38^, ^39^, ^40^, ^41^, ^42^, ^43^, ^44^, ^45^, ^46^, ^47^, ^48^, ^49^, ^50^ Histological assessments encompassed collagen fiber organization, cellularity, nuclear morphology, inflammatory infiltrates, and Bonar scores.^18^, ^19^, ^20^, ^21^, ^23^, ^24^, ^32^, ^33^, ^37^, ^41^, ^44^, ^45^, ^46^, ^50^

Functional outcomes were systematically categorized to represent distinct mechanical domains. Load‐bearing capacity was quantified through tensile strength, ultimate stress, ultimate load, breaking strength, and load at break.^15^, ^20^, ^22^, ^23^, ^27^, ^29^ Elastic and deformation properties were assessed using Young's modulus, stiffness, ultimate strain, extension at break, and energy to failure.^15^, ^27^, ^29^ Functional resistance and mobility were evaluated based on adhesion scores, joint activity, stretches, and the forces applied.^23^, ^27^, ^35^ This categorization enabled a comprehensive and structured assessment of tendon mechanical recovery.

Overall, PBM showed wavelength‐ and dose‐dependent effects, enhancing collagen organization, modulating inflammatory mediators, and improving tendon mechanical performance across both acute rupture and chronic tendinopathy models.

### Molecular Outcome

PBM reduced *IL‐1β*, *IL‐6*, tumor necrosis factor α, reactive oxygen species, and thiobarbituric acid reactive substance in acute tendon rupture models, mitigating inflammation and extracellular matrix disorganization.^16^, ^25^, ^37^ Both LLLT and PTB achieved comparable antioxidative effects, with Fillipin et al.^16^ and Yao et al.^25^ reporting significant reductions in oxidative stress after 14 to 21 days and 200 s of exposure, respectively.

In the proliferative phase, PBM enhanced fibroblast activity and angiogenesis through increased vascular endothelial growth factor expression^26^ and transient downregulation of tenogenic markers (*SCX*, *Tnmd*), indicating earlier remodeling.^33^ During remodeling, PBM promoted macrophage transition from M1 to M2 phenotypes and modulated *TGF‐β1* expression, accompanied by elevated *IL‐10* and a higher collagen I/III ratio.^21^, ^30^, ^31^, ^33^

These molecular effects in **acute tendon injury models** were confirmed quantitatively through meta‐analysis (Figures 2 and 3). Across 4 studies (n = 44 tendons), PBM significantly reduced *IL‐1β* compared with controls (MD = −46.14 pg/mL; 95% CI − 66.45 to −25.84; *P* < .00001)—Figure 2—whereas 3 studies (n = 36 tendons) showed a consistent increase in *IL‐10* (MD = 49.14 pg/mL; 95% CI 37.41‐60.86; *P* < .00001)—Figure 3. Heterogeneity was high for *IL‐1β* (*I* ^2^ = 85%) but negligible for *IL‐10* (*I* ^2^ = 0%), reflecting consistent cytokine upregulation across studies using LLLT wavelengths between 660 and 904 nm. Subgroup analysis showed comparable anti‐inflammatory effects between laser‐based PBM (LLLT) and LED PBM, with no significant subgroup difference for either cytokine.

In **chronic tendinopathy models**, PBM suppressed *IL‐1β*, *COX‐2*, *PGE_2_*, reactive oxygen species, and *MMPs*,^1^, ^3^, ^13^, ^19^ while upregulating *TGF‐β1* and vascular endothelial growth factor, facilitating collagen type I restoration.^34^, ^35^, ^36^, ^38^, ^39^, ^40^, ^41^, ^42^, ^45^, ^46^, ^49^ De Jesus et al.^47^ noted that overdosing (17.5 J/cm^2^) negated these effects, supporting a biphasic dose‐response.

### Histological Outcome

PBM‐induced molecular modulation translated into clear histological improvements. In acute tendon rupture, PTB offered a nonsurgical method to preserve collagen integrity.^51^ Ding et al.^23^ found that PTB at 532 nm and 200 J/cm^2^ significantly reduced inflammatory cell infiltration compared with suture repair at days 7 and 14, accompanied by lower apoptosis, denser collagen fibers, and organization resembling native tendon tissue.^24^

In chronic tendinopathy, PBM restored matrix alignment and normalized collagen composition. Laser irradiation at 830 nm increased type I collagen predominance at days 7 to 21, outperforming both control and diclofenac‐treated groups.^42^, ^46^ Vedda et al.^50^ observed improved histoarchitecture with 660 and 808 nm wavelengths, with 808 nm producing superior collagen organization. Similarly, LED PBM at 880 nm (7.5 J/cm^2^) reduced inflammatory infiltration and fibroblast activity while enhancing fiber production and realignment.^38^

Bonar score analysis supported progressive structural recovery, with PBM groups approaching near‐normal values by day 21.^19^ Da Ré Guerra et al.^37^ reported early extracellular matrix modulation, including decreased noncollagenous proteins and glycosaminoglycans and transient *MMP‐9* upregulation within 4 h postinjury. Evangelista et al.^44^ further noted enhanced neovascularization, collagen alignment, and reduced necrosis in PBM‐treated tendons.

Meta‐analysis (Figure 4) confirmed these histological findings. Five studies (n = 72 tendons) showed a significant increase in the collagen I/III ratio at **day 14** after PBM compared with control (MD = 0.79; 95% CI 0.67‐0.92; *P* < .00001), indicating enhanced collagen type I predominance during tendon remodeling. Subgroup analysis showed similar effects for LLLT (MD = 0.79; 95% CI 0.66‐0.93; *I* ^2^ = 0%) and LED PBM (MD = 0.80; 95% CI 0.36‐1.24; *P* = .98), with minimal heterogeneity (*I* ^2^ = 0%), confirming consistency across modalities.

### Functional Outcomes

Tendon repair aims to restore mechanical strength and functional integrity. Eight studies evaluated biomechanical outcomes—6 on tendon rupture^15^, ^20^, ^22^, ^23^, ^27^, ^29^ and 2 on tendinopathy^36^, ^49^—using parameters such as tensile strength, Young's modulus, and rupture resistance. Across rupture models, PBM consistently improved tensile strength compared with conventional repair or untreated controls, while preserving joint mobility and reducing fibrotic adhesion.^23^, ^27^

Chan et al.^22^ reported superior biomechanical restoration with PTB compared with LLLT, and Ding et al.^23^ showed that PTB combined with human amniotic membrane enhanced gliding and reduced postoperative adhesions versus suture repair. Combining LLLT with platelet‐rich plasma further increased tensile strength,^20^ whereas Locke et al.^15^ found LLLT alone insufficient for complete structural recovery. All PTB studies employed **sutureless photochemical bonding**. In Ding et al.,^23^ a temporary alignment suture was removed intraoperatively, confirming that all PTB models represented nonsuture photochemical bonding rather than surgical augmentation.

In tendinopathy, PBM improved failure force, stiffness, and stress tolerance.^36^, ^49^ Haslerud et al.^49^ also found greater force and displacement when cryotherapy was combined with LLLT.

Meta‐analysis (Figures 5 and 6) confirmed these findings. Pooled Newton‐based data^15^, ^20^, ^29^, ^49^ showed a significant increase in tensile strength favoring PBM (MD = 10.07 N; 95% CI 3.01‐17.13; *P* = .005; *I* ^2^ = 87%). MPa‐based data^22^, ^23^, ^27^ showed that PTB significantly improved load‐bearing strength (MD = 0.57 MPa; 95% CI 0.37‐0.78; *P* < .00001), confirming enhanced early mechanical stabilization through photochemical collagen cross‐linking.

### Risk of Bias

**FIGURE 7:**
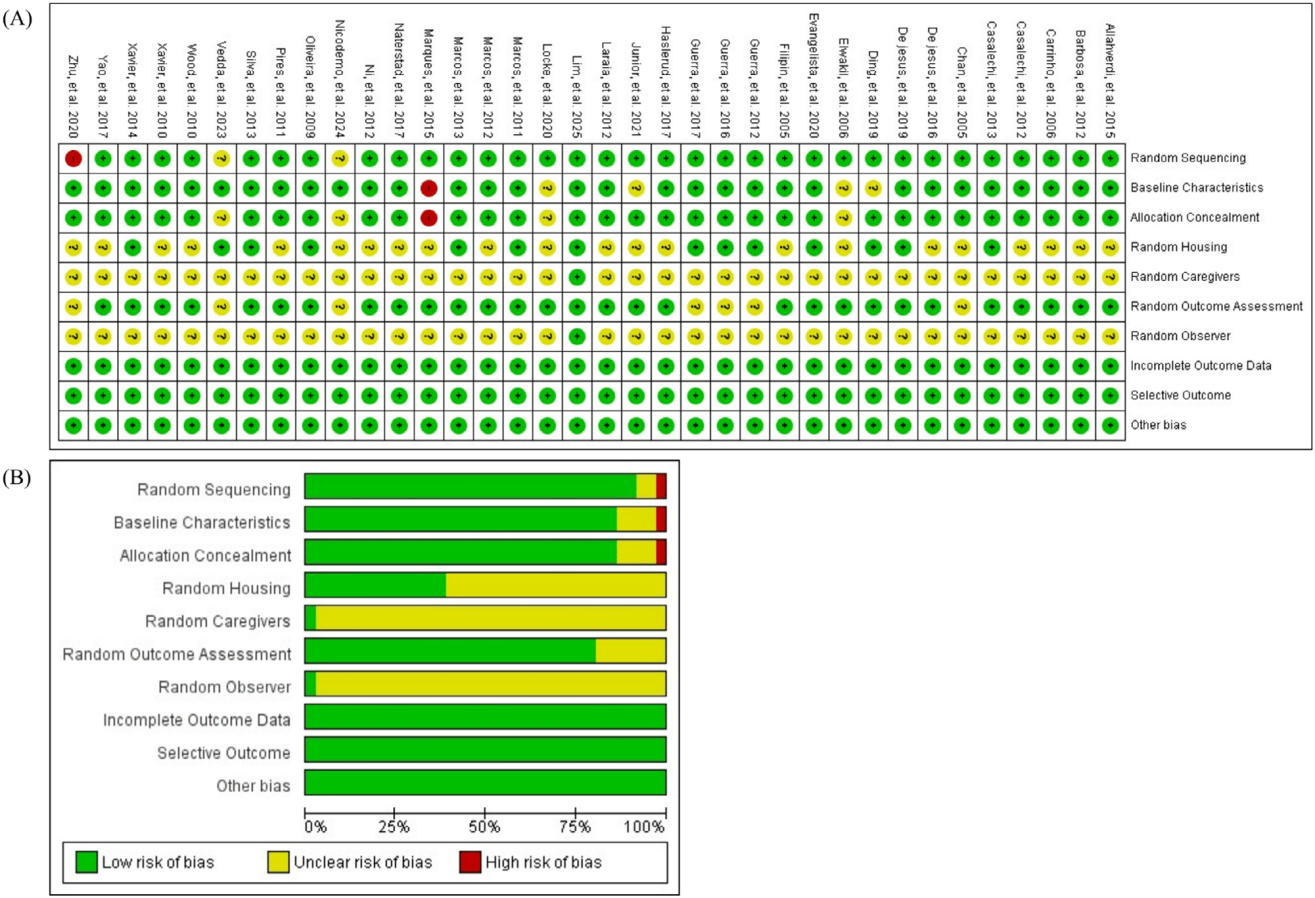
Risk of bias summary (A) and domain‐level graph (B) based on the SYRCLE tool across the included animal studies. Green indicates low risk, yellow represents unclear risk, and red denotes high risk of bias across various methodological domains, including randomization, blinding, and outcome report. (SYRCLE, Systematic Review Centre for Laboratory Animal Experimentation.)

The quality assessment of 36 animal and laboratory studies was reported using the summary risk of bias (Figure 7). Two studies had a high risk of bias, whereas the remaining studies had unclear risk.^24^, ^46^ Due to insufficient information, most studies had an unclear risk of bias regarding random housing, caregivers, and observers.

Funnel plot analysis (Figures S1‐S5) showed overall symmetry across all pooled outcomes, suggesting a low risk of publication bias. Minor asymmetry observed in the Newton‐based functional outcome was attributed to variability in irradiation parameters and load measurement methods rather than selective reporting. The MPa‐based PTB, histological, and molecular outcomes (IL‐1β and IL‐10) showed balanced distributions, supporting the consistency and reliability of the included studies. As each meta‐analysis included fewer than 10 studies, these plots were interpreted descriptively in accordance with Cochrane recommendations.

## DISCUSSION

Quantitative synthesis showed that PBMs exert consistent regenerative effects across molecular, histological, and functional domains in preclinical tendon models (see Figures 2, 3, 4, 5, 6). PBM reduced IL‐1β by approximately 40%, increased IL‐10 by 65%, enhanced the collagen type I/III ratio by 1.7‐fold, and improved tensile strength by about 20% compared with untreated controls. These multidomain effects collectively indicate that PBM accelerates inflammatory resolution, promotes collagen remodeling, and restores biomechanical integrity of the tendon, supporting the hypothesized photobiological mechanism of regeneration.

### Molecular Modulation

PBM enhances mitochondrial activity through photon absorption by cytochrome c oxidase, increasing adenosine triphosphate synthesis and displacing nitric oxide inhibition.^26^ This mechanism aligns with the meta‐analytic findings of reduced *IL‐1β* and elevated *IL‐10* in acute models (Figures 2 and 3), confirming PBM's dual anti‐inflammatory and immunomodulatory roles.

These cytokine shifts promote macrophage polarization from proinflammatory M1 to reparative M2 phenotypes via *IL‐10* and *TGF‐β1* signaling,^21^, ^33^ facilitating inflammation resolution and collagen remodeling.^30^, ^31^, ^33^ In chronic tendinopathy, PBM reactivates tenocytes and restores extracellular matrix turnover, reversing low‐grade inflammation and collagen type III predominance.^52^

Additionally, PBM‐induced expression of HSP70 enhances cellular defense against oxidative and apoptotic stress,^44^ strengthening tissue resilience during tendon regeneration. Collectively, these molecular mechanisms provide a biologic rationale for the improved histological organization and functional recovery shown across studies.

### Histological Remodeling

PBM promotes collagen maturation and fiber realignment through mitochondrial activation and regulation of matrix turnover. The increased collagen I/III ratio observed in meta‐analysis (Figure 4) substantiates these effects, indicating enhanced synthesis of type I collagen and accelerated remodeling.^19^, ^23^, ^24^, ^37^, ^38^, ^42^, ^44^, ^46^, ^50^

Mechanistically, PBM regulates fibroblast proliferation and extracellular matrix remodeling via *TGF‐β1* and *MMP‐9* signaling pathways.^37^, ^38^ The comparable outcomes between LLLT and LED PBM suggest wavelength‐independent stimulation of fibroblast and angiogenic activity, consistent with findings by Vedda et al.^50^ and Evangelista et al.^44^ In acute rupture, PTB yielded superior collagen continuity without sutures,^23^, ^24^, ^27^ whereas in tendinopathy models, LLLT and LED PBM improved collagen density, alignment, and neovascularization.

Collectively, these results confirm PBM's capacity to accelerate tendon remodeling by reestablishing collagen organization, reducing inflammation, and promoting matrix regeneration through light‐mediated cellular bioactivation.

### Functional Recovery

PBM enhances the biomechanical competence of healing tendons by coupling cellular bioactivation with collagen realignment. The meta‐analytic increase in load‐bearing strength (in Newtons) confirms PBM's efficacy in enhancing tendon mechanical resilience, whereas PTB improved intrinsic tensile strength (in MPa) through collagen cross‐linking. These complementary mechanisms reflect PBM's role in functional strengthening and PTB's role in microstructural stabilization.

LLLT achieves this effect via mitochondrial stimulation and growth factor modulation, promoting fibroblast proliferation and myofibroblast contractility.^15^, ^20^, ^49^ PTB, in contrast, restores mechanical continuity through photochemical collagen bonding, enabling sutureless repair while maintaining tissue gliding and elasticity.^22^, ^23^, ^24^ The incorporation of human amniotic membrane or platelet‐rich plasma further enhances mechanical outcomes by augmenting biological scaffolding and cytokine support.^20^, ^23^

Despite heterogeneity in wavelength and energy density, the consistent tensile improvements across models underscore PBM's robust regenerative and mechanical effects. Collectively, these results highlight PBM's translational potential as a noninvasive adjunct that accelerates tendon repair and restores functional capacity through both structural and molecular mechanisms.

### Limitations

This study is not without limitations. Only 1 study assessed sex‐related effects with nonsignificant results, whereas most did not report sex, limiting generalizability given its potential impact on tendon healing.^15^ Considerable heterogeneity in PBM parameters and outcome measures reduced study comparability. A partial meta‐analysis of homogeneous subgroups was conducted; however, inconsistent reporting and variable quality limited the broader quantitative synthesis. Most studies showed unclear risk of bias, and all were restricted to animal or laboratory models, which constrains direct clinical applicability.

## CONCLUSIONS

PBMs promote tendon regeneration in both acute and chronic injury models, enhancing tendon regeneration at molecular, histological, and functional levels.

## SUPPORTING INFORMATION

Additional supporting information can be found online in the Supporting Information section.

## DECLARATION OF GENERATIVE AI AND AI‐ASSISTED TECHNOLOGIES IN THE WRITING PROCESS

During the preparation of this work, the authors used ChatGPT (OpenAI, San Francisco, CA, USA) to improve the clarity and language of the manuscript. After using this tool, the authors reviewed and edited the content as needed and take full responsibility for the content of the publication.

## DISCLOSURES

The authors (Y.W., D.N.I.M., M.N.A., Y.R., M.S.A.A.) declare that they have no known competing financial interests or personal relationships that could have appeared to influence the work reported in this article.

## Supporting information

Supplementary Material
