# Supplementary material for: Photobiomodulation Enhances Tendon Regeneration: A Systematic Review and Meta‐analysis of Preclinical Studies
Source: Arthrosc Sports Med Rehabil. 2026 Jul 8;8(4):e70029. doi: 10.1002/ars2.70029 (PMC13399650; doi:10.1002/ars2.70029)
Supplement: Supplementary file 1 [file ARS2-8-e70029-s001.pdf]

Supporting Information

Figure S1. Funnel plot assessing publication bias for studies evaluating the effect of photobiomodulation on pro-inflammatory IL-1β levels.

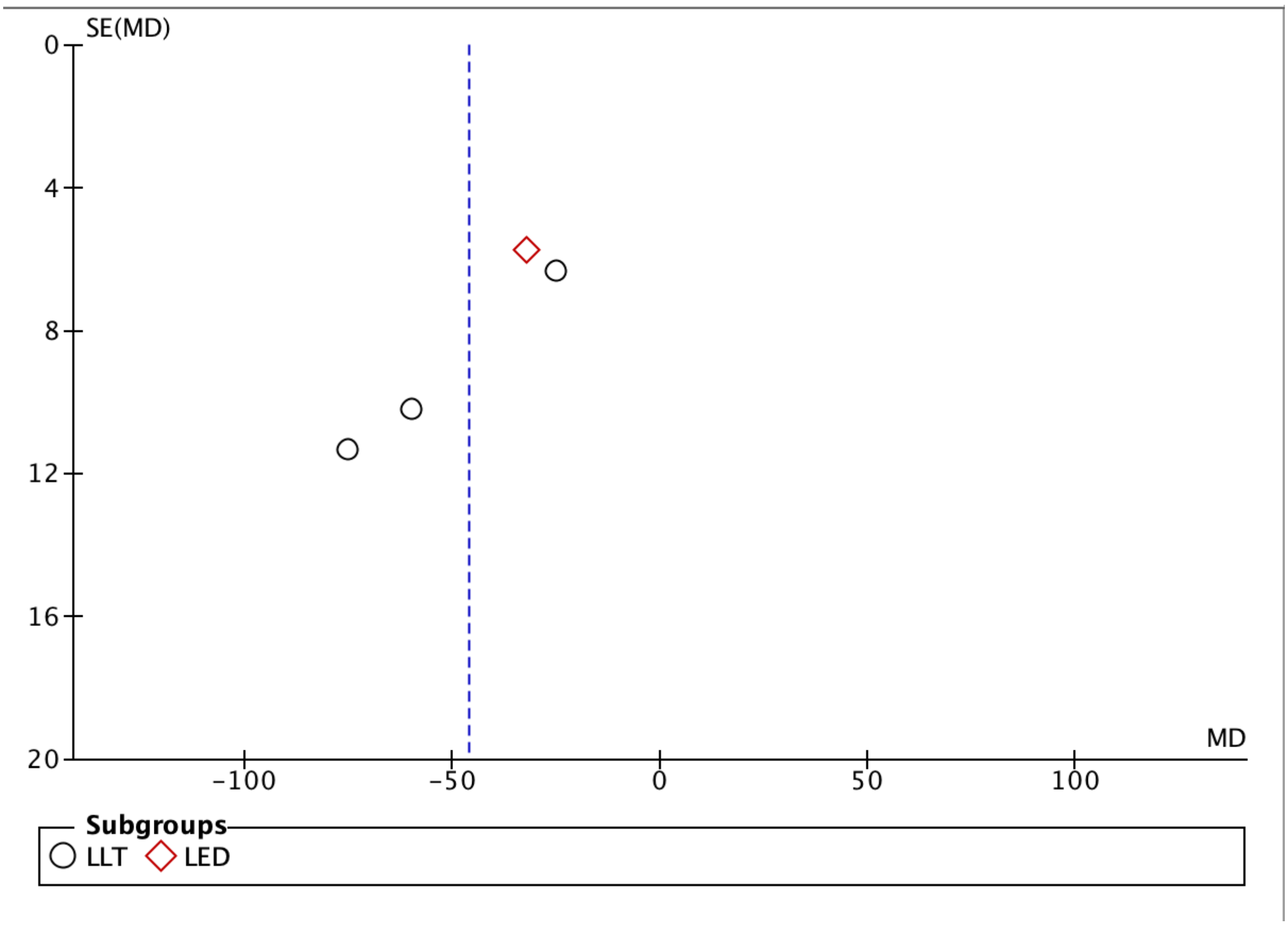

Figure S2. Funnel plot assessing publication bias for studies evaluating the effect of photobiomodulation on anti-inflammatory IL-10 levels.

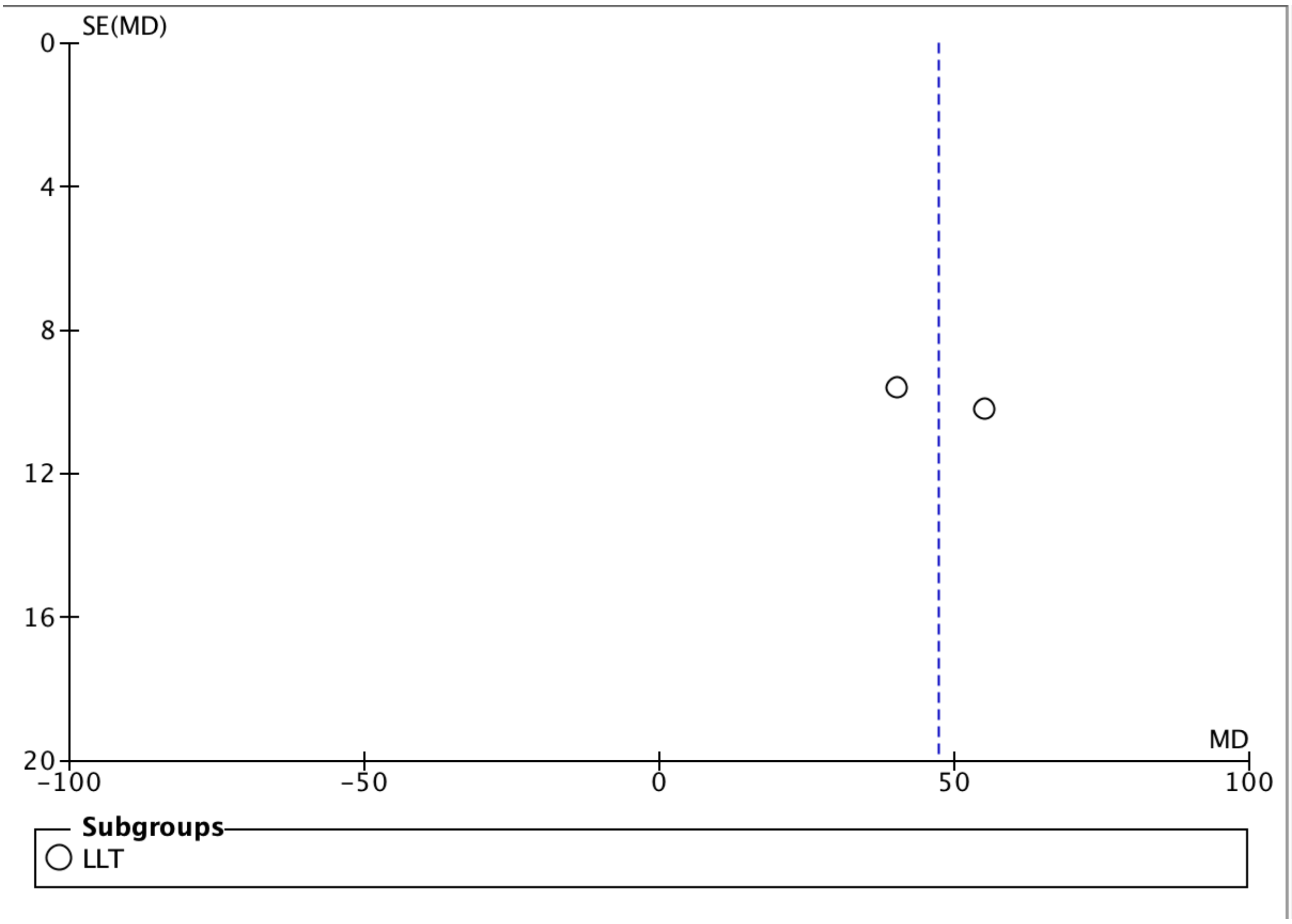

Figure S3. Funnel plot assessing publication bias for histological outcomes (collagen type I/III ratio).

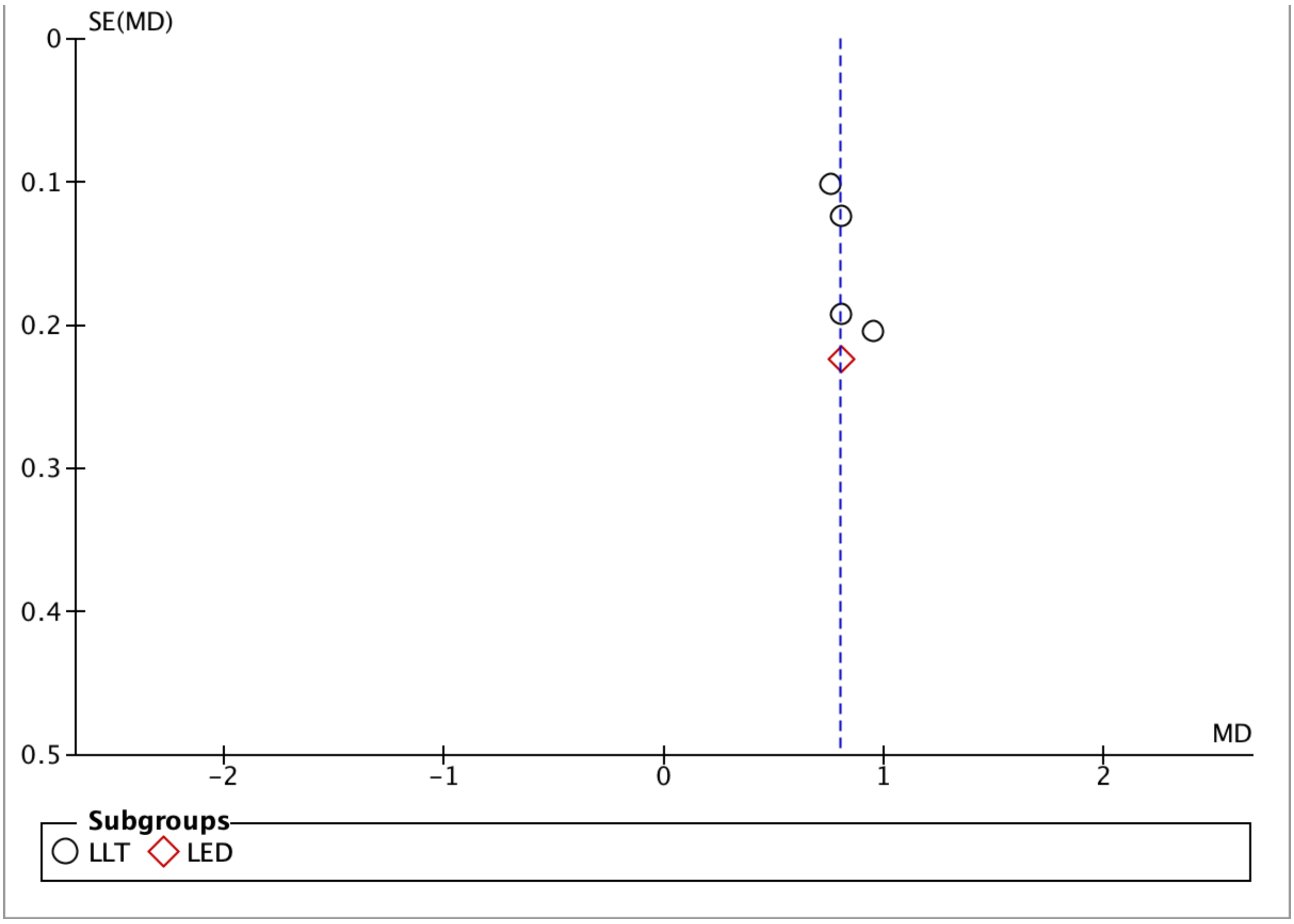

Figure S4. Funnel plot assessing publication bias for functional outcomes (load-bearing strength, N) in photobiomodulation (LLLT and LED) studies.

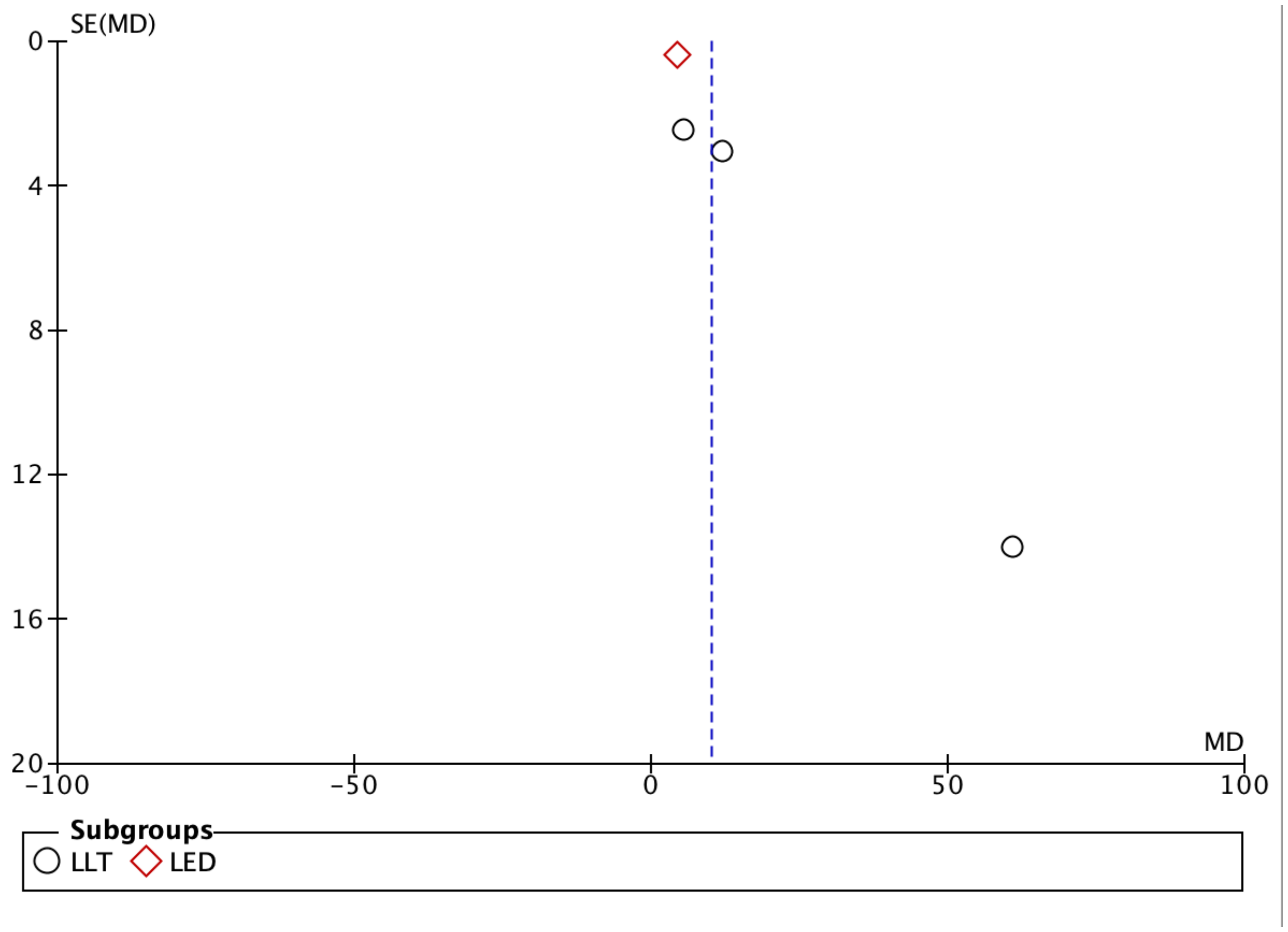

Figure S5. Funnel plot assessing publication bias for functional outcomes (tensile strength, MPa) in photochemical tissue bonding (PTB) studies.

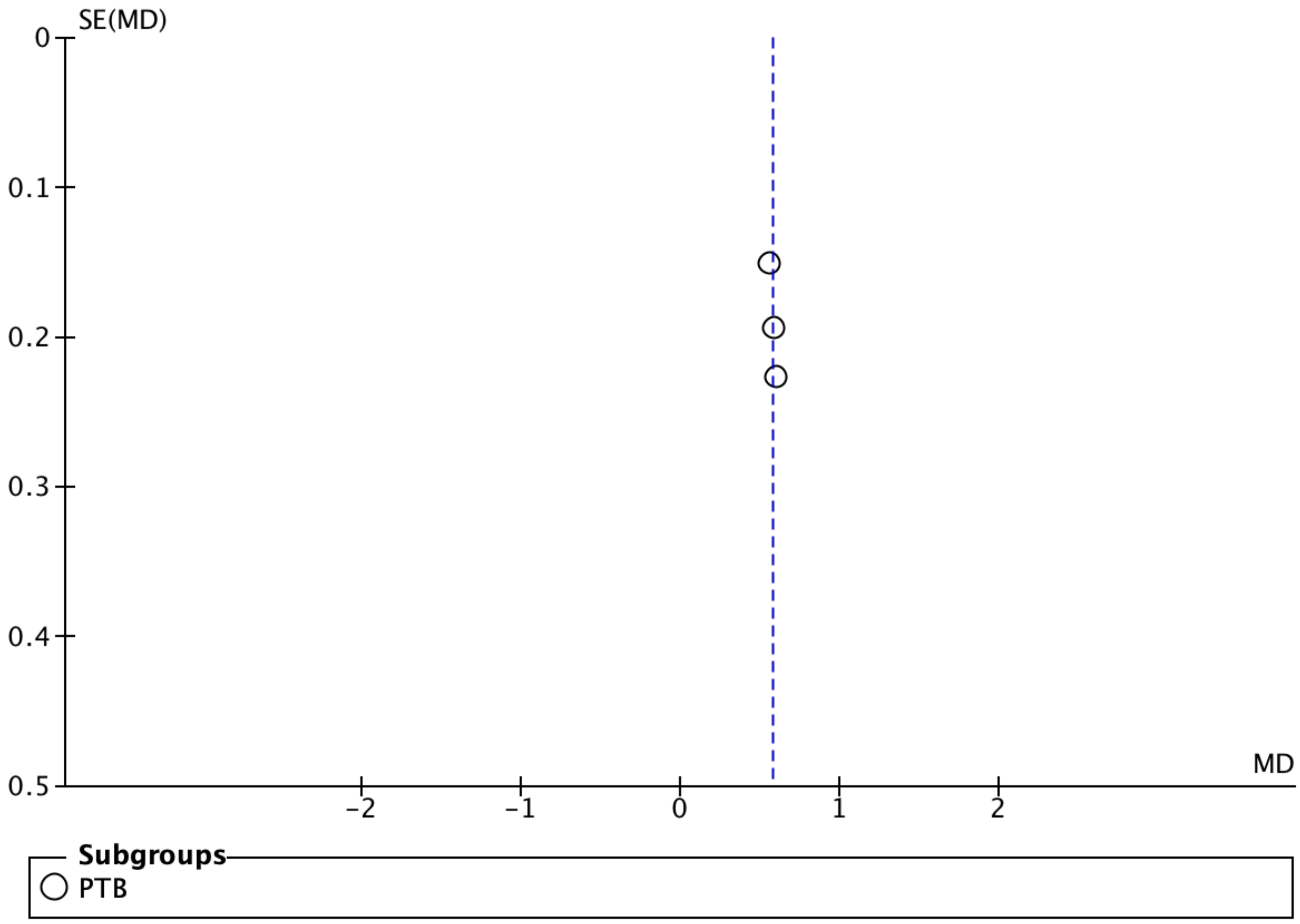
SE(MD)
0
0.1
0.2
0.3
0.4
0.5
MD
−2
−1
0
1
2
Subgroups
PTB
